# Identification of allergens and epitopes involved in allergy to deamidated gluten

**DOI:** 10.1186/2045-7022-3-S3-O16

**Published:** 2013-07-25

**Authors:** S Denery, C Brossard, C Larré, M Bodinier, F Pineau, M Pietri, DA Moneret-Vautrin, E Paty

**Affiliations:** 1 UR 1268 BIA Nantes, INRA, Nantes, France; 2 Service d'allergologie, Centre Hospitalier Jean Monnet, Epinal, France; 3 Service de Pneumologie et Allergologie Pédiatriques, Groupe Hospitalier Necker, Paris, France

## Background

Gluten proteins can be modified by deamidation to enhance their solubility and technological applications. However severe allergic reactions have been reported after the consumption of food products containing deamidated gluten in subjects tolerant to wheat. This work aimed to characterize allergen profiles for these patients in comparison with those of patients allergic to wheat and identify IgE-binding epitopes.

## Methods

Sera were obtained from 15 patients allergic to deamidated gluten (DG) and from 9 patients allergic to wheat proteins (WP). IgE-binding profiles were characterized both in ELISA and in a humanized rat basophilic leukemia (RBL) cell model. Epitopes were mapped on γ and ω2-gliadin sequences by Pepscan and effect of glutamine/glutamic acid substitutions were studied.

## Results

Compared to the heterogeneous pattern of allergens detected by IgE from patients allergic to WP, responses of patients allergic to DG were homogeneous. In ELISA, all the sera displayed IgE-binding to deamidated γ and ω2-gliadins and deamidated total gliadins, frequently with high concentrations. These modified proteins induced RBL degranulation with most of the sera from DG-allergic patients. A consensus epitope was found on native γ and ω2-gliadins (QPQQPFPQ); it was repeated several times in their sequences. The substitution of two or three glutamines of this epitope into glutamic acid at positions Q3 or Q4 and Q8 (QPEEPFPE) increased its recognition the best.

## Conclusion

Allergy to DG is a separate entity from wheat allergy characterized by a homogeneous IgE response. Deamidated ω2-gliadins or the dominant IgE-binding epitope QPEEPFPE could be used as tools for the diagnosis of this new allergy.

## Disclosure of interest

None declared.
